# Wanted—Co-Operation

**Published:** 1911-04-08

**Authors:** 


### The Hospital

A Journal of

The Medical Sciences and Hospital Administration.

New Series. No. 218, Vol. IX. [No. 1290, Vol. L.] SATURDAY, APRIL 8, 1911

### WANTED—CO-OPERATION.

Now and again in the history of nations there occur occasions which are the opportunities that make statesmen as distinct from politicians. The clash of opposing forces stilled for a brief moment, the evolution of new factors, the sudden, meteoric flash of popular opinion, the quick seizure of all or any of these moments serve to turn incidents into events and to demonstrate the difference between a Cambacérès and a Napoleon. The one, too keen on his cookery, neglected the opportunities which his position as consul gave him for the reformation of the hospital system in France; the other, great in social no less than in military strategy, routed the forces of organised opposition and called into being a hospital and sick relief service which for its time was worthy the reputation of the Emperor and the indefatigable Larrey. A similar opportunist, in the best sense of the term in which opportunism connotates statesmanship, was Bismarck when he created the system of State insurance that exists in the German Empire. Napoleon's hospitals are now historical expressions merely; and Bismarck's scheme has developed into a monster which urgently needs control and bridling, but the fact remains that both have been of incalculable benefit to the countries in which they were born and that both have immensely helped to educate the public in social matters.

To any thoughtful person who at the present moment contemplates the position of hospitals in this country, and regards simultaneously the trend of public opinion, it is obvious that we stand on the eve of great alterations. Intentionally we do not say developments, for the nature and beneficial consequences of these modifications of our hospital system must depend entirely on the manner in which the great problem of reconstruction is handled. It is equally evident that there is no public man in Parliament among us who has paid so much and such thorough attention to the matter that he will be able to play Napoleon or Bismarck for us during the next few years when the subject of poor and sick relief must engage consideration from any Government and any Opposition. It is true we have Cambacérès, not enslaved by his gastrosophical speculations, but absorbed and prejudiced by his view of side issues: Cambacérès in various disguises: the national medical service man, the minority man, the State hospital man, the conservative voluntaryist, and the hierarchical professional man. And what we want is a constructive statesman who can take advantage of the present opportunity to mould and direct public opinion in the best interests of our charitable hospitals.

That the opportunity is there, no one who is acquainted with hospital work and politics during the last quarter of a century can doubt. We are all agreed that an alteration, a modification, is desirable, though we are not all unanimous on the point of the reason for such change, and still less with regard to the form it must take. Mr. Walter Long, in his recent speech at York, and Sir Henry Burdett, in his letters to the Press dealing with that pronouncement, and the address to the Home Hospitals Association (which is fully reported in this issue), have both drawn attention to the necessity for change: in this journal we have given many articles, during the last five years, presenting the arguments for and against such modifications in all their various aspects. On all sides there is a manifest desire to touch the subject, but the pity is that in many quarters there is no appreciation of the great and diverse difficulties which lie in the way of true reconstruction, and often not even a sense of the real nature of things as they are. The nonsense talked at some recent meetings in London, for example, whereat the voluntary system and the King's Fund were denounced as the greatest stumbling blocks in the way of reform, is a proof of the fact that the general public does not yet understand the ins and outs of the hospital problem. In these circumstances it is of the greatest importance that public men who are interested—as they ought to be interested—in the problem should co-operate with hospital workers in order to get the information and facts which they require to deal with the problem in a comprehensive fashion, with the object of promoting the ideal system—a thoroughly modern medical and nursing service for all classes of the community. This is a matter which affects the public as much as it does the institutions, and the initiative may as fittingly come from the former as from the latter. It does not matter from whom it emanates so long as it comes quickly. The present state of indecision is regrettable in every respect. We shall soon have to embark on an extended policy of reform and it is highly desirable that we shall do so thoroughly prepared to adopt the best solution of the problem that presents itself. There is just a chance that we may be cajoled, owing to the efforts of an ignorant but vociferous section, into creating a worse system, which will need reconstruction in a very few years hence. What we require is co-operation, not merely in the final scheme, whereby every charitable agency in the kingdom shall be a unit in a correlated system of charitable relief, but in the preliminaries: a close co-partnership between workers and speakers, institutional and public men.

